# Sequencing technology as a major impetus in the advancement of studies into rheumatism: A bibliometric study

**DOI:** 10.3389/fimmu.2023.1067830

**Published:** 2023-02-17

**Authors:** Runzhi Huang, Jieling Tang, Siqiao Wang, Yifan Liu, Mengyi Zhang, Minghao Jin, Hengwei Qin, Weijin Qian, Yuwei Lu, Yiting Yang, Bingnan Lu, Yuntao Yao, Penghui Yan, Jie Huang, Wei Zhang, Jianyu Lu, Minyi Gu, Yushu Zhu, Xinya Guo, Shuyuan Xian, Xin Liu, Zongqiang Huang

**Affiliations:** ^1^ Department of Orthopedics, The First Affiliated Hospital of Zhengzhou University, Zhengzhou, China; ^2^ Department of Burn Surgery, The First Affiliated Hospital of Naval Medical University, Shanghai, People’s Republic of China, Research Unit of Key Techniques for Treatment of Burns and Combined Burns and Trauma Injury, Chinese Academy of Medical Sciences, Shanghai, China; ^3^ Shanghai Jiao Tong University School of Medicine, Shanghai, China; ^4^ Tongji University School of Medicine, Shanghai, China; ^5^ Department of Orthopedics, Shibei Hospital, Shanghai, China; ^6^ Department of Rheumatology and Immunology, Second Affiliated Hospital of Naval Medical University, Shanghai, China

**Keywords:** rheumatism, sequencing, expression, classification, identification

## Abstract

**Background:**

Rheumatism covers a wide range of diseases with complex clinical manifestations and places a tremendous burden on humans. For many years, our understanding of rheumatism was seriously hindered by technology constraints. However, the increasing application and rapid advancement of sequencing technology in the past decades have enabled us to study rheumatism with greater accuracy and in more depth. Sequencing technology has made huge contributions to the field and is now an indispensable component and powerful tool in the study of rheumatism.

**Methods:**

Articles on sequencing and rheumatism, published from 1 January 2000 to 25 April 2022, were retrieved from the Web of Science™ (Clarivate™, Philadelphia, PA, USA) database. Bibliometrix, the open-source tool, was used for the analysis of publication years, countries, authors, sources, citations, keywords, and co-words.

**Results:**

The 1,374 articles retrieved came from 62 countries and 350 institutions, with a general increase in article numbers during the last 22 years. The leading countries in terms of publication numbers and active cooperation with other countries were the USA and China. The most prolific authors and most popular documents were identified to establish the historiography of the field. Popular and emerging research topics were assessed by keywords and co-occurrence analysis. Immunological and pathological process in rheumatism, classification, risks and susceptibility, and biomarkers for diagnosis were among the hottest themes for research.

**Conclusions:**

Sequencing technology has been widely applied in the study of rheumatism and propells research in the area of discovering novel biomarkers, related gene patterns and physiopathology. We suggest that further efforts be made to advance the study of genetic patterns related to rheumatic susceptibility, pathogenesis, classification and disease activity, and novel biomarkers.

## 1 Introduction

Rheumatic diseases, that is, autoimmune and inflammatory diseases in which the immune system attacks joints, muscles, bones, and organs, causing chronic pain, and having obscure etiology, have plagued humans for thousands of years (1). Although the initial identification of rheumatism can be traced as far back as AD 123, when Indian doctor Charaka Samhita first recorded rheumatism symptoms (2), for many years our understanding of rheumatic diseases was seriously hindered by technology constraints and the variety of clinical manifestations in the diseases. However, during the past decades, thanks to the advancement of sequencing technology and important discoveries made in immunology and molecular biology, studies into rheumatic diseases have taken a huge leap. With such developments, a more comprehensive perspective of rheumatism has unfolded.

Rheumatism encompasses a wide range of diseases (3). According to the American Rheumatism Association nomenclature and classification of arthritis and rheumatism, published in 1983, rheumatism can be further categorized into (1) diffuse connective tissue diseases, e.g., rheumatoid arthritis, lupus erythematosus, and scleroderma; (2) arthritis associated with spondylitis, e.g., ankylosing spondylitis; (3) osteoarthritis (i.e., degenerative joint disease); (4) rheumatic syndromes associated with infectious agents, e.g., acute rheumatic fever; (5) metabolic and endocrine diseases associated with rheumatic states, e.g., gout; (6) neoplasms; (7) neurovascular disorders, e.g., Charcot joint and spinal stenosis; (8) bone and cartilage disorders, e.g., osteoporosis and osteomalacia; (9) extra-articular disorders, i.e., juxta-articular lesions and miscellaneous pain syndromes; and (10) miscellaneous disorders associated with articular manifestations, e.g., palindromic rheumatism. However, this classification is still under revision because of emerging evidence, driven by the development of technology and analytical tools. Of the over 200 diseases which qualify as rheumatic diseases, several have attracted particular attention. (4) Rheumatoid arthritis (RA), the prevalence of which is highest in northern and western Europe (0.4%), showed possible increasing trends for occurrence (5). Systemic lupus erythematosus (SLE), a heterogeneous disease that typically affects women from 16 to 55 years old, based on the research of European Reference Networks (ERN) on rare and complex connective tissue and musculoskeletal diseases (ERN ReCONNET) (6), has a global prevalence of 13 to 7,713.5 per 100,000 individuals (7). Osteoarthritis (OA), the most common joint disorder (8), is estimated to affect an estimated 240 million individuals worldwide (9), sometimes with severe complications, such as osteoporosis (1). OA and chronic inflammatory diseases such as RA and SLE (10) are among the rheumatic diseases most commonly studied, in fields from molecular biology (11) to regenerative medicine (12).

Sequencing technology, that is, the technology that detects the sequence pattern of biomacromolecules in the central dogma of molecular biology. covers DNA sequencing to RNA sequencing to protein sequencing and provides us with insights into the relationship between genotypes and phenotypes (13). It laid down a solid foundation of bioinformatics and now is branching into subfields with great potential, such as the transcriptomic, proteomic, and metabolomic subfields (14). Sanger sequencing first made complete genome sequence deciphering possible 40 years ago. Since then, a second revolution has taken place, with the appearance of next-generation sequencing technologies that are cheaper and quicker. After the completion of the Human Genome Project in 2000 and the appearance of next-generation sequencing in 2004, the era of post-genomics dawned, with the focus shifting to transcriptomics and proteomics, to study dynamic gene expression with higher speed and more accuracy. Genome-wide association studies (GWASs), whose first results were reported in 2005 and 2006, allowed many discoveries into the susceptibility of diseases with genetic patterns (15). Moreover, the development of single-cell sequencing technology, since its establishment in 2009, has allowed for a more precise analysis of the heterogeneity of cells (16) and is an effective tool in the dissection of complex cellular events, such as immune responses, especially single-cell RNA sequencing (17). In recent years, technologies with higher precision in long reads and the detection of epigenetic modifications have emerged, ushering sequencing technology into its third revolution (18).

The progress made in sequencing technologies has been echoed in the recently improved understanding of rheumatism. The advancement and widened application of sequencing technology and bioinformatics has helped us to take a deeper look into rheumatic diseases on their molecular and cellular levels, and has become an indispensable research tool for its great contributions to the classification, pathogenesis, diagnosis, and treatment of rheumatic diseases. GWASs could be used to explore associations between genetic variants and phenotypes (19). The increasing application of GWASs in research in rheumatism has promoted the identification of important genetic variants associated with susceptibility of rheumatism. Single-cell RNA sequencing technology made exciting breakthroughs in the differentiation and identification of synovial cells, presenting new therapeutic targets and remarkable changes in treatment (20); mass spectrometry imaging has also been performed on articular cartilage, synovium, and bone. This has further expanded our knowledge of articular destruction and enhanced the characterization of diagnostic and prognostic biomarkers for osteoarthritis, rheumatoid arthritis, and osteoporosis (21).

Bibliometrics takes the global document characteristics and literature landscape as its research object and utilizes methods that include statistics and mathematics to explore the quantitative relationships, changing laws, and distribution patterns of document information, thereafter the characteristics, structures, and laws of technology and science are analyzed. (22), which could assist researchers in better and more quickly grasping the course, trend, and current hotspots of a specific field. In addition, it can identify reliable researchers and affiliations for cooperation (23). More and more scholars are applying bibliometrics to guide studies, such as in cardiovascular diseases (24), cancer (25), and even during the COVID-19 pandemic (26). In recent years, research related to sequencing technologies in rheumatism has grown rapidly, but no studies have used bibliometrics and visualization approaches to perform deep mining and explore the field in detail. Hence, it is necessary to conduct bibliometrics analysis to sort and analyze the published research to get a quick overview and find meaningful research directions. Accordingly, to fill this gap, in this study we performed a systematic bibliometric analysis to describe the current research situation in the field, including information on the countries contributing the most, the most relevant authors and affiliations,, and so on. In addition, by analyzing the most frequently used keywords and their co-occurrence, we identified the hottest and significant themes in the published literature. Based on the analysis of current literature, we provide a roadmap for spotting changes in focus and emerging trends in future studies of rheumatism sequencing.

## 2 Methods

### 2.1 Data sources and retrieval strategies

The Web of Science™ (Clarivate™, Philadelphia, PA, USA), the most trusted global database, and the strongest search engine, was used for publication retrieval on 25 April 2022. The retrieval strategy was as follows: (((TS=rheumatology) OR (TS=rheumatic disease) OR (TS=rheumatism)) AND ((TI=transcriptomic) OR (TI=proteome) OR (TI=proteomics) OR (TI=metabolomics) OR (TI=bioinformatics) OR (TI=metagenomics) OR (TI=metatranscriptomics) OR (TI=omics) OR (TI=microarray) OR (TI=sequence) OR (TI=RNA-seq) OR (TI=sequencing) OR (TI=ATAC-seq) OR (TI=single cell sequencing) OR (TI=single cell sequence) OR (TI=single cell RNA sequencing) OR (TI=single cell RNA sequence) OR (TI=expression profile) OR (TI=bioinformatic*) OR (TI=high throughput))). We retrieved a total of 1,374 articles published from 1 January 2000 to 25 April 2022 in the Web of Science Core Collection. Reviews and monographs were excluded as publication types, and articles, meeting abstracts, proceedings papers, editorial material, letters, and corrections were retained (**Figure S1**). Altogether, 1,374 publications of these types were considered as relevant articles in this bibliometric study.

### 2.2 Data analysis

All data were exported in a text file and were analyzed using Bibliometrix (version 3.2.1), an open-source tool developed by Massimo Aria and Corrado Cuccurullo using statistical computing and R language (version 4.2.0, Institute for Statistics and Mathematics, Vienna, Austria; www.r-project.org) for quantitative research in scientometrics. Bibliometrix can support the workflow for bibliometric analysis and can be easily upgraded and integrated with other R packages (The R Foundation for Statistical Computing, Vienna, Austria) (27). Other than Bibliometrix, Citespace (5.8.R3) and VOSviewer (v.1.6.15, Centre for Science and Technology Studies, Leiden University, Leiden, the Netherlands) were also widely applied in this bibliometrics study. Developed by Dr Chaomei Chen using Java, CiteSpace can construct and assess co-citation networks *via* visualization analysis (28). VOSviewer, capable of generating a co-occurrence matrix from keywords (29), was also very popular among researchers. In the present study, countries, authors, affiliations, co-cited publications, and co-occurrence of keywords were analyzed. The dimensionality reduction technique was used to visualize the conceptual structure of keywords to identify the frontier studies and hotspots.

## 3 Results

### 3.1 Annual analysis of publications

**Figure 1:**
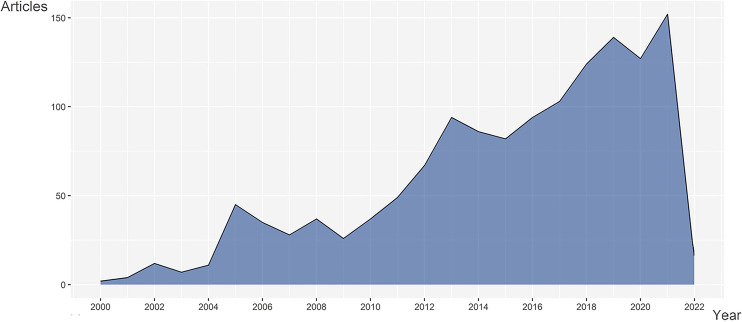
Analysis of annual output of articles on rheumatism and sequencing.

Since the year 2000, publications in rheumatism sequencing have shown an overall tendency of growth. From 2000 to 2004, few articles were published in this area. In 2005, the number of publications increased in line with the development of proteomics and maturation of microarrays. After 2009, with the emergence of single-cell sequencing, publication numbers rapidly increased, with more effort dedicated to the field using this technology. The data retrieval date (April 25) may account for the steep decline in 2022 (**Figure 1**). Taken together, these results indicate that this field is attracting more and more attention and has great potential for future development.

### 3.2 Countries, authors, and affiliations

**Figure 2:**
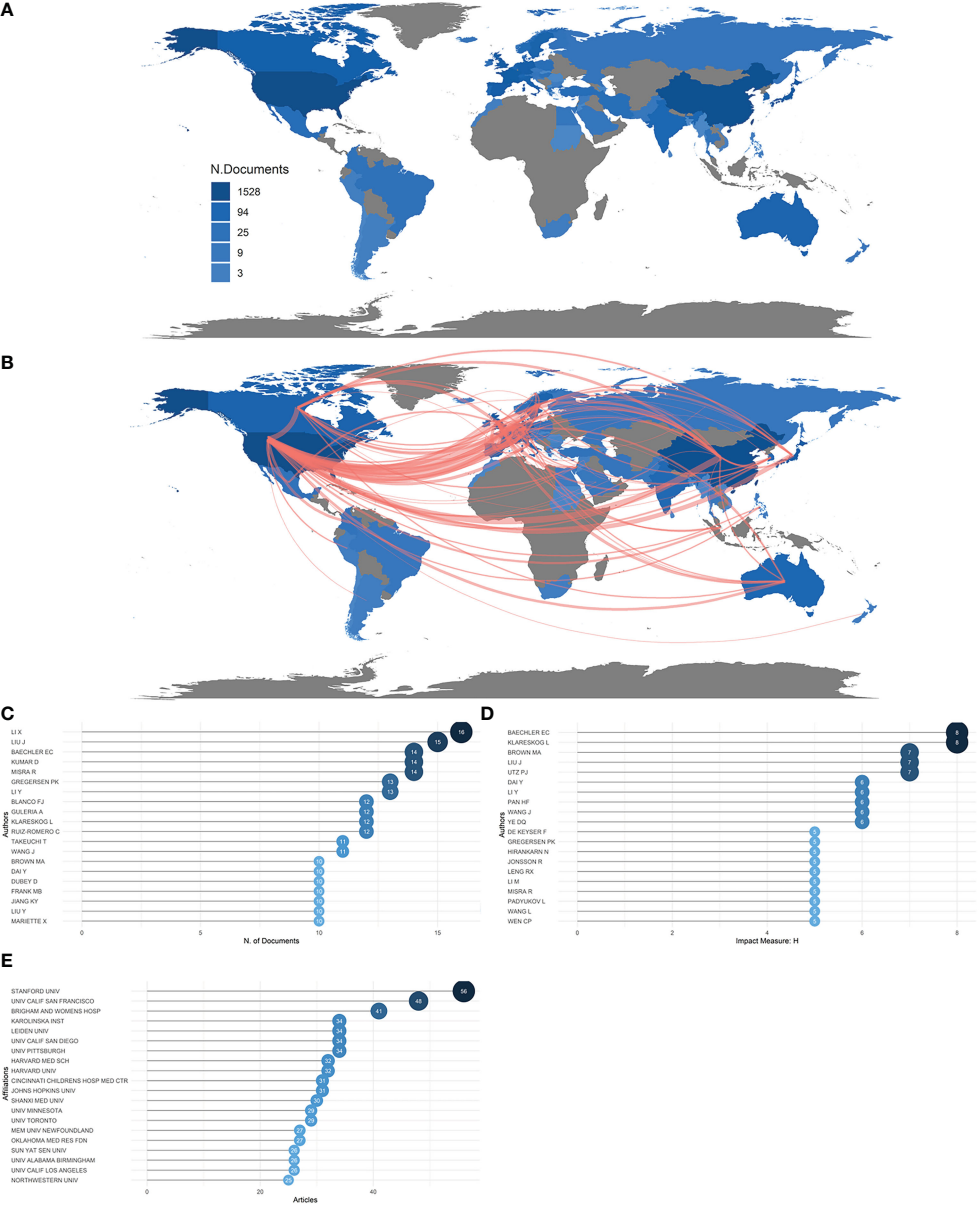
Countries, authors, and institutions. **(A)** Country scientific production map. The darker the color, the greater the number of documents (frequency) published by a country or region. **(B)** Country collaboration map. Red lines represent the collaboration bonds between countries. The thicker the line, the stronger the collaboration between countries. **(C)** Most relevant authors in the field of rheumatism and sequencing. The size and darkness of the nodes are in proportion to the number of documents published by a particular author. **(D)** Most influential authors, as measured by the h-index. The size and darkness of the nodes are in proportion to the h-index for each author. **(E)** Most relevant affiliations in the field of rheumatism and sequencing. The size and darkness of the nodes are in proportion to the number of documents produced by the affiliation.

A total of 1,374 articles were retrieved, from 62 countries, led by the USA and China, which accounted for 334 publications and 300 publications, respectively, creating a huge production gap between these two countries and the rest of the world. Ranked by accumulated publication frequency, the USA came first, with 1,592 publications, and China second, with 982 publications (**Figure 2A**). In addition, China and the USA were the two most cited countries across all publications, indicating excellence in both the quantity and quality of their research (**Figure S2A**). The top 20 most productive countries are listed in **Table 1**, with the USA, China, Japan, Germany, South Korea, United Kingdom, Australia, Sweden, Italy, and India making up the top 10. Collaboration strength between countries can be inferred from the single-country publication (SCP) and multiple-country publication (MCP) rates. The countries that had the highest MCP ratio were Switzerland, the United Kingdom, the Netherlands, Canada, and Norway, that is, mostly European countries; other countries, such as China, Korea, and Belgium, focused more on domestic publication. As shown in **Figure 2B**, the USA had strong cooperative bonds with China, Korea, Canada, and European countries in particular, whereas China has strong bonds with the USA, Europe, and Korea. Although the USA’s and China’s MCP ratios were not the highest, they still ranked first and second, respectively, for total number of MCPs (**Figure S2B**).

**Table 1:** The top 20 most productive countries in rheumatism and sequencing studies.

Rank	Country	Articles (*n*)	Frequency	SCP		MCP	MCP ratio
1	USA	334	0.325219	257		77	0.231
2	China	300	0.292113	262		38	0.127
3	Japan	46	0.044791	36		10	0.217
4	Germany	34	0.033106	25		9	0.265
5	South Korea	29	0.028238	26		3	0.103
6	United Kingdom	24	0.023369	10		14	0.583
7	Australia	22	0.021422	17		5	0.227
8	Sweden	21	0.020448	13		8	0.381
9	Italy	20	0.019474	15		5	0.25
10	India	19	0.0185	13		6	0.316
11	Netherlands	18	0.017527	9		9	0.5
12	Canada	16	0.015579	8		8	0.5
13	Spain	13	0.012658	11		2	0.154
14	France	12	0.011685	10		2	0.167
15	Poland	10	0.009737	8		2	0.2
16	Norway	9	0.008763	5		4	0.444
17	Belgium	8	0.00779	7		1	0.125
18	Iran	8	0.00779	6		2	0.25
19	Switzerland	8	0.00779	0		8	1
20	Denmark	7	0.006816	5		2	0.286

MCP, multiple-country publication; SCP, single-country publication.

During the last 22 years, a number of authors have played active roles and made great efforts to push the field forward. Lotka’s law describes the pattern between authors and their number of published papers (30). According to Lotka, the number of authors with *n* published articles is 1/*n* ^2^ of those with one published article (31). In general, this law describes the phenomenon that a small number of authors produce a large proportion of relevant articles. This law also applies to publications in rheumatism and sequencing, as a large proportion of the publications in rheumatism and sequencing were published by a rather small proportion of authors (**Figure S2C**). The top 20 most productive authors are listed in **Figure 2C**, with Li, Liu, Baechler, Kumar and Misra accounting for the top 5. Judging by the h-index, Baechler and Klareskog were the most productive authors, with the highest H-index, of 8, meaning that they produced eight documents, all with an impact factor above 8. They were followed by Liu, Utz , and Dai, with an h-index of 7 (**Figure 2D**). However, the number of studies produced by Baechler reached its peak in 2005, and he ceased to produce studies altogether after 2017, whereas Kumar, although starting late, in 2015, has retained an energetic role until today. Li, Liu, Kumar, Misra, Blanco,Guleria, Takeuchi, Wang, Dai, Dubey, Liu, and Mariette continued to be active after 2020. (**Figure S2D**).

The source of authors’ inspiration, and the incubator for their discoveries, is their institution. Thus, it is of great importance to identify the prolific institutions. **Figure 2E** shows that Stanford University was positioned first, with 56 publications, followed by the University of California, San Francisco (*n* = 48), Brigham and Women’s Hospital (*n* = 41), the Karolinska Institute (*n* = 34), Leiden University (*n* = 34), and the University of California, San Diego (*n* = 34), four of which are in the USA. Shanxi Medical University, the affiliation of the most prolific author, Li, and the University of Minnesota, the affiliation of the third most prolific author, Baechler, also made the top 20. These results are in line with the accumulated production figures for countries noted above, indicating that the USA is in the vanguard of the field.

### 3.3 Source analysis

**Figure 3:**
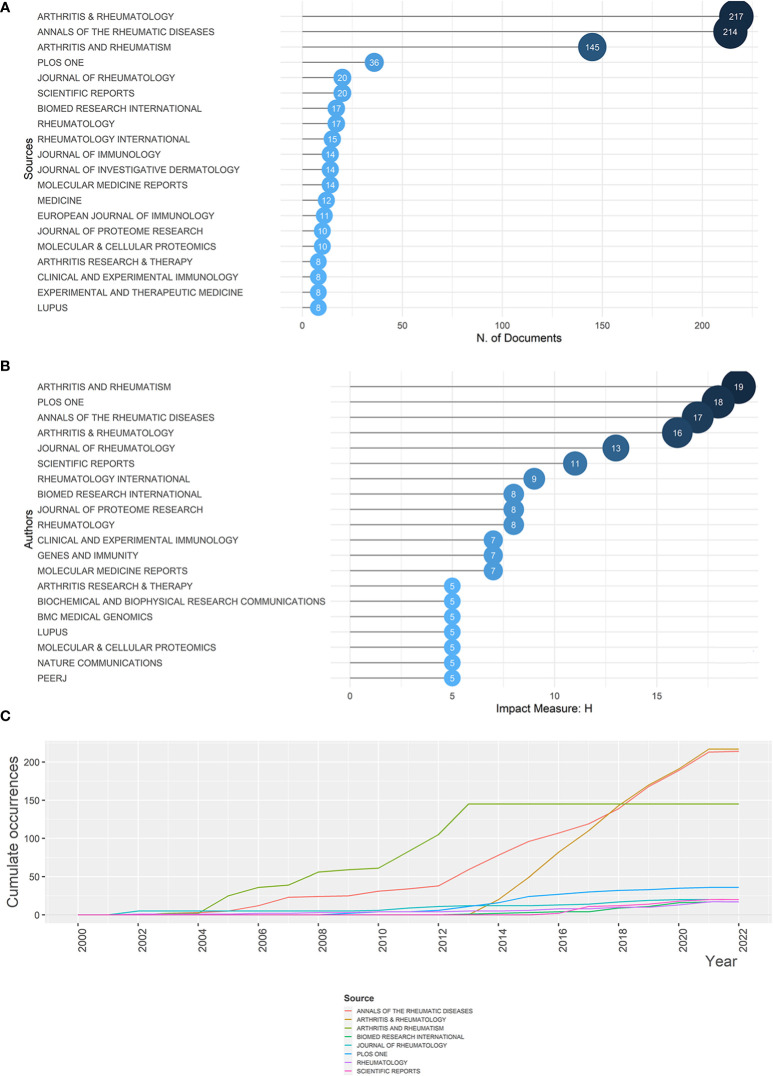
Most relevant sources for rheumatism and sequencing and their growth. **(A)** Top 20 most relevant sources for rheumatism and sequencing. The diameter and darkness of the node are in proportion to the number of documents published by the source. **(B)** Sources’ growth from 2000 to 2022. **(C)** The top 20 sources with the highest h-index. The diameter and darkness of the node are in proportion to the h-index of the source.

Since the year 2000, 1,374 articles have been published, from 350 sources. The top 20 most popular journals are illustrated in **Figure 3A**. Arthritis & Rheumatology (which was named as Arthritis & Rheumatism before 2014), *Annals of the Rheumatic Diseases*, and *Arthritis & Rheumatism* were the top three, all with publications numbers surpassing 140, accounting for the majority of the relevant articles (**Figure S3**). During the last 22 years, cumulate co-occurrences in all journals presented an increase, among which *Arthritis & Rheumatology* and *Annals of the Rheumatic Diseases* displayed a rapid increase after 2012 or 2013, signaling an upsurge in the importance [or popularity of these journals. *Arthritis & Rheumatism*, on the other hand, manifested a stable level after growing from 2004 to 2013. Detailed trends are presented in **Figure 3B**. Furthermore, taking the impact of journals into account, *Arthritis & Rheumatism* ranks in first place, with an H-index of 19, followed by *PLOS ONE* (H = 18), *Annals of the Rheumatic Diseases* (H = 17), and *Arthritis & Rheumatology* (H = 16), making them valuable sources for research related to rheumatism and sequencing (**Figure 3C**).

### 3.4 Cited documents and references

**Figure 4:**
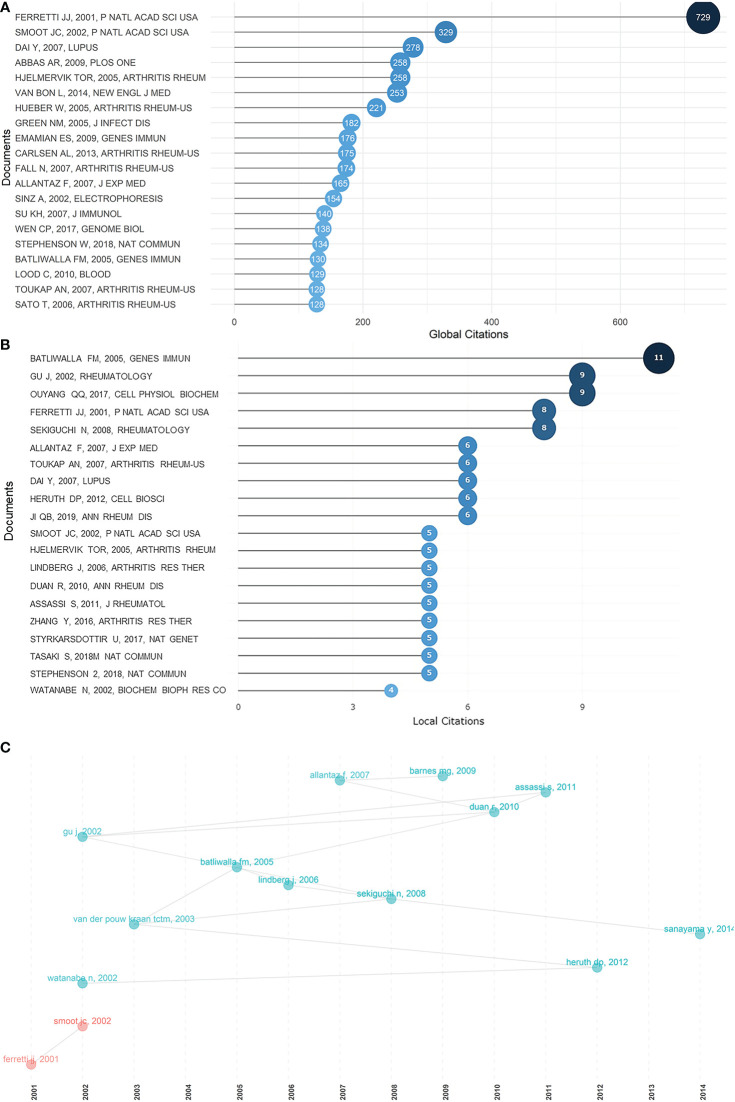
Most cited documents in rheumatism and sequencing, and their correlations. **(A)** Top 20 most globally cited documents concerning rheumatism and sequencing. The size and darkness of the nodes are in proportion to the number of global citations of each document. **(B)** Top 20 most locally cited documents concerning rheumatism and sequencing. The size and darkness of the nodes are in proportion to the number of local citations of each document. **(C)** Historical direct citation network. Each dot represents a document and is labeled with first author’s surname and the publication year.

Certain documents or references cited laid served as future directions for research into rheumatism. Globally cited documents are those that are cited in the Web of Science database, whereas locally cited documents are those that are cited in this collection of articles. Thus, high numbers of globally cited documents indicated overall influence, whereas high numbers of locally cited documents suggest an impact in this field. **Figures 4A**, **B** show the top 20 globally cited documents and the top 20 locally cited documents. The most globally cited document and fourth most locally cited document, “Complete genome sequence of an M1 strain of *Streptococcus pyogenes”* (32), by Ferretti in 2001, revolutionarily identified the genes responsible for “molecular mimicry” causing rheumatic fever or acute glomerulonephritis, and set an inspiring premise for subsequent research. It was cited in the paper “Genome sequence and comparative microarray analysis of serotype M18 group A *Streptococcus* strains associated with acute rheumatic fever outbreaks” (33) by Smoot in 2002 (**Figure 4C**), which is also the second most globally cited document, digging deeper into the molecular basis of acute rheumatic fever pathogenesis. “Peripheral blood gene expression profiling in rheumatoid arthritis” (34), by Batliwalla, published in 2005, was first among the most locally cited documents, and was also in the top 20 most globally cited documents, providing the groundwork of gene expression patterns of rheumatoid arthritis. “Gene expression profile analysis of rheumatoid synovial fibroblast cultures revealing the overexpression of genes responsible for tumor-like growth of rheumatoid synovium” (35), by Watanabe, published in 2002, “A 588-gene microarray analysis of the peripheral blood mononuclear cells of spondyloarthropathy patients” (36), by Gu, published in 2002, and “Discovery of distinctive gene expression profiles in rheumatoid synovium using cDNA microarray technology: evidence for the existence of multiple pathways of tissue destruction and repair” (37), by van der Pouw Kraan, published in 2003, were three pioneering articles in the exploration of rheumatoid pathogenesis. Among the most locally cited references (**Figure S4A**), “The American Rheumatism Association 1987 revised criteria for the classification of rheumatoid arthritis” (38), by Arnett, ranked first, revealing the central and fundamental role of the definition and classification of rheumatism. The dominant position of this article can also be seen in the Sankey diagram (**Figure S4B**). Ranking the second in the most locally cited references was “Interferon-inducible gene expression signature in peripheral blood cells of patients with severe lupus” (39), by Baechler, published in 2003, which widened our knowledge of SLE. Baechler's work showed a high level of inheritance, that is, novel findings were derived from his own previous research.

Factorial analysis in bibliometric studies can identify a smaller group of factors that describe the correlations between the input literature, and form clusters according to the interrelationships among them (40). Articles that appear closer on the figure share stronger correlations. Applying factorial analysis, these most cited documents can be broken down into distinct clusters. In consonance with the historical direct citation network, the work of Smoot distributed in the vicinity of Ferretti, confirming their strong interrelationship (**Figure S5A**). As shown in in **Figure S5B**, the documents with the highest contributes, “Perturbations in amino acids and metabolic pathways in osteoarthritis patients determined by targeted metabolomics analysis” (41) by Chen, “Comparative analysis of synovial fluid and plasma proteomes in juvenile arthritis—Proteomic patterns of joint inflammation in early stage disease” (42) by Gibson, “Proteomics analysis for verification of rheumatoid arthritis biomarker candidates using multiple reaction monitoring” (43) by Lee, and “Proteomics in rheumatology” (44) by Kabeerdoss, scattered near each other. These four documents share a common focus, that of proteomics, demonstrating the significance of proteomics in the study of rheumatism. Relatively far from the clusters lies the work of Xie, “Bioinformatics analysis of epigenetic and SNP-related molecular markers in systemic lupus erythematosus” (45), which might indicate the emergence of epigenetics in rheumatism research.

### 3.5 Hotspots and frontiers

**Figure 5:**
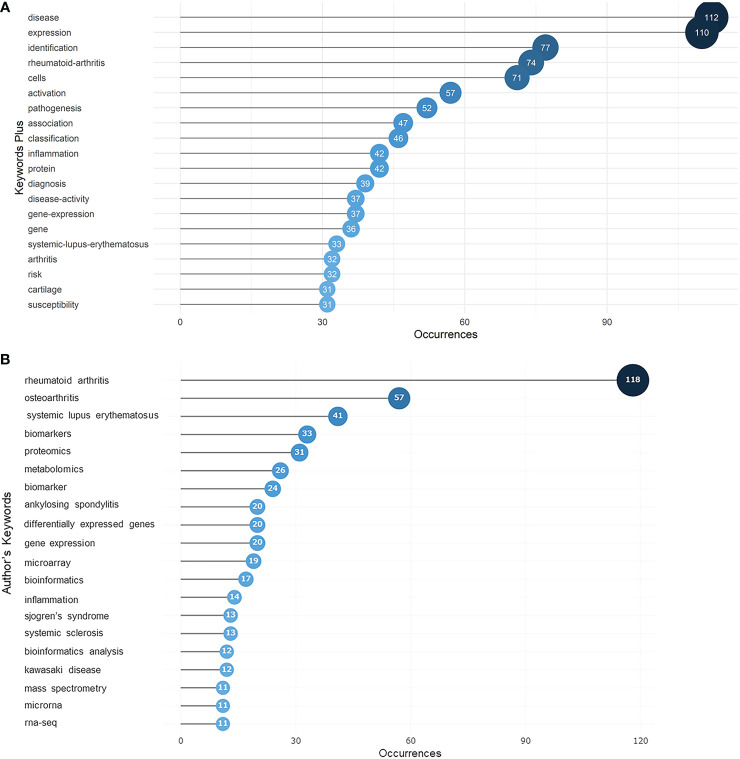
Ocuurences of keywords in articles regarding rheumatism and sequencing determined by KeyWords Plus analysis. **(A)** Top 20 most frequent keywords selected from KeyWords Plus. The size and darkness of the nodes are in proportion to the number of occurrences of each keyword. **(B)** Top 20 most frequent keywords supplied by authors. The size and darkness of the nodes are in proportion to the number of occurrences of each keyword. Thematic evolution shown in the Sankey diagram from 2000 to 2022.

Keywords that constantly appeared in selected articles reflect the hotspots for research to some extent. KeyWords Plus (Clarivate) are words or phrases that are calculated by special algorithms that do not appear in the title of the article but appear in the titles of the article’s references. Article keywords, in contrast, are supplied by the article authors themselves. The KeyWords Plus analysis (**Figure 5A**) revealed that “disease” and “expression” were the two most common keywords among the articles we examined with 112 and 110 occurrences, respectively, far exceeding all other keywords, implying that the field was disease oriented, and focused on dynamic gene expression instead of just sequencing. This high frequency of appearance of “disease” and “expression” was visualized using a keyword tree and word cloud (**Figures S6A, B**). Based on our analysis of authors’ keywords (**Figure 5B**), it is evident that most work was dedicated to rheumatoid arthritis, followed by rheumatism, osteoarthritis, and systemic lupus erythematosus.

**Figure 6:**
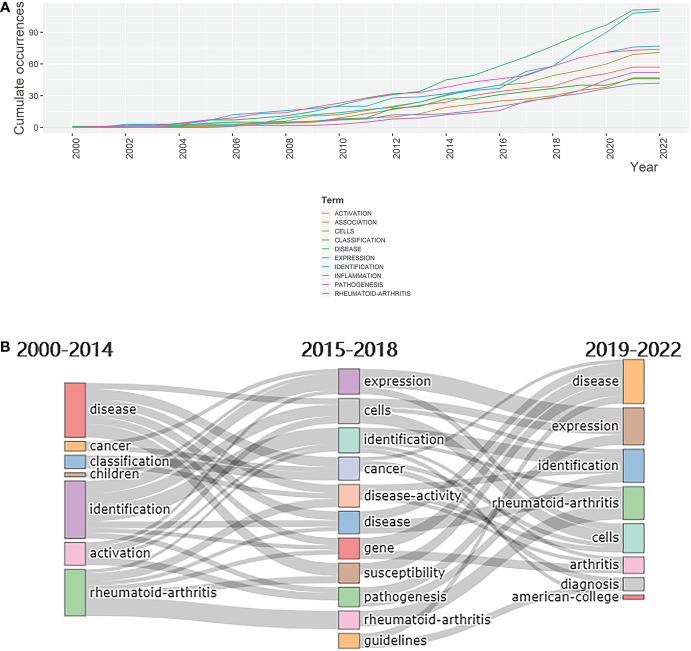
Evolution of keywords. **(A)** Evolution, from 2000 to 2022, of the keywords shown in the Sankey diagram.**(B)** Dynamic changes in keywords’ cumulative occurrences from 2000 to 2022.

In addition to determining the frequency of keywords, it is also of great importance to investigate their evolution and development. Over the period from 2000 to 2022, all selected keywords displayed an increasing level of occurrence. Among all keywords, “disease” and “expression” showed robust growth, indicating promising future development (**Figure 6A**). The Sankey diagram of keywords in which time is divided into the periods 2000–2014, 2015–2018, and 2019–2022 (**Figure 6B**) shows that “disease”, “identification”, and “rheumatoid-arthritis” attracted the most attention during the first decade of the century. The focus then shifted to keywords such as “expression”, “cells”, “cancer”, “gene”, “susceptibility”, and “pathogenesis”. However, in the most recent years, that is from 2019 to 2022, “disease” and “identification” regained their popularity among researchers. In addition, “rheumatoid-arthritis”, one of the best-known rheumatic diseases, has consistently been a focus for researchers over the last 22 years. It can be deduced from this diagram that sequencing technology mainly served as tools for the study of rheumatic diseases and their mechanisms. The prevalence of the word “expression” suggests the growing attention paid to transcriptomes, in turn reflecting the increasing value of sequencing technology. The emergence of “cells” may imply that is becoming associated more closely with studies investigating rheumatism on a cellular level is becoming associated more closely with studies investigating rheumatism on a cellular level. The value of exploring novel biomarkers *via* sequencing technology is confirmed by the emergence of “identification”.

**Figure 7:**
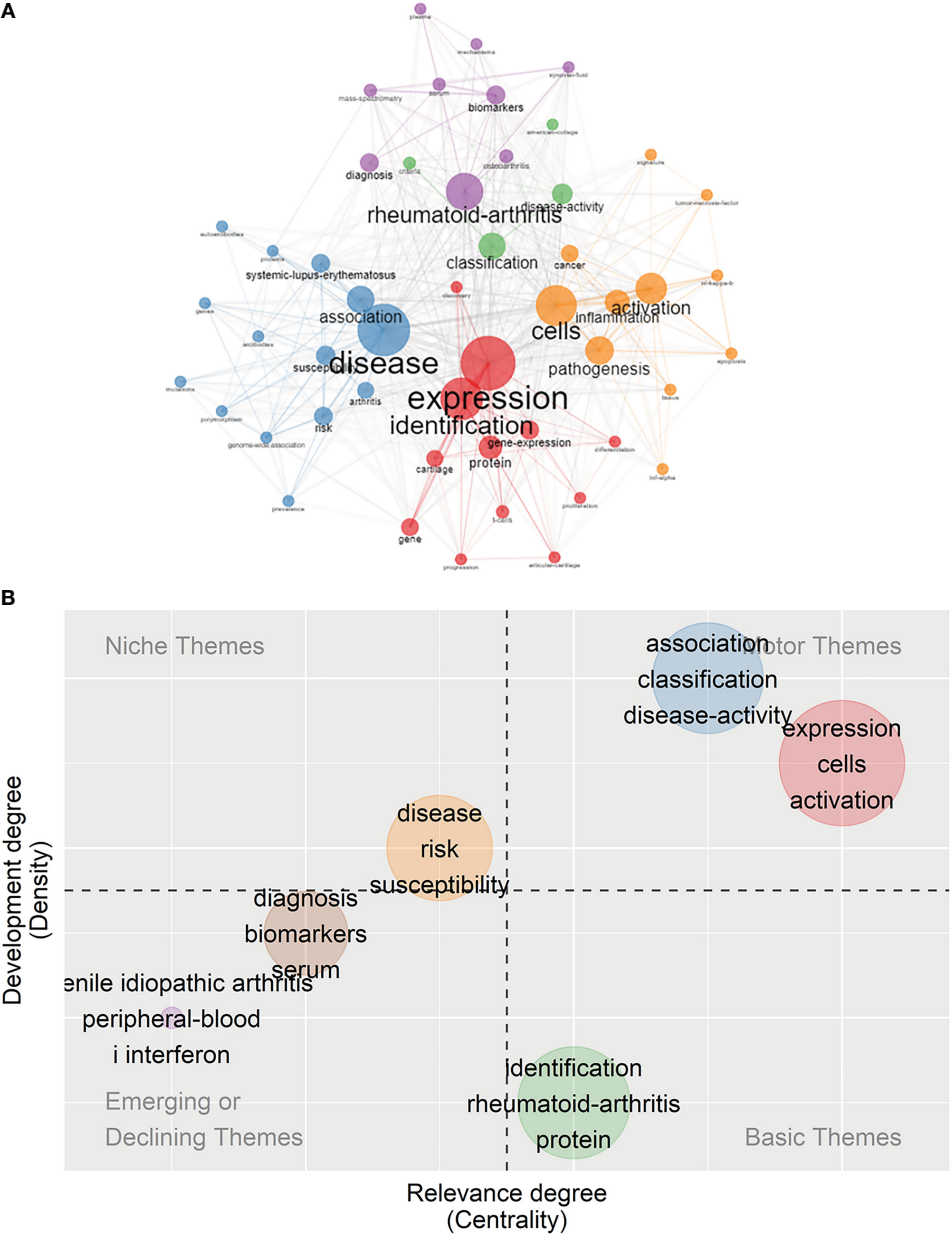
Co-word analysis and prospects of themes. **(A)** Co-occurrence analysis of keywords. Keywords were divided into five clusters labeled with different colors (blue, red, orange, green, and purple). **(B)** Thematic map of themes. The five themes are each labeled with three keywords chosen to be representative of the theme.

In addition to identifying the popular words, it is crucial to analyze the co-words network to make out the conceptual structure. Keywords were divided into five clusters, as illustrated in **Figure 7A**, and words belonging to the same cluster were given the same color. The blue cluster mainly consisted of the terms “disease”, “association”, “susceptibility”, “risk”, “arthritis”, “systemic-lupus-erythematosus”, and “genome–wide association”. The red cluster included the terms “expression”, “identification”, “gene-expression”, “protein”, “cartilage”, “articular-cartilage”, “gene-expression”, and “gene”. The orange cluster was composed of the terms “cells”, “inflammation”, “activation”, “pathogenesis”, and “apoptosis”. The green cluster comprised the terms “classification” and “disease-activity”. The purple cluster contained the words “rheumatoid-arthritis”, “diagnosis”, “osteoarthritis”, “biomarkers”, “serum”, “mass-spectrometry”, and “synovial-fluid”. Diameters of the nodes were in proportion to the occurrences, and the line thickness that bridged nodes together indicated co-occurrences.

Applying factorial analysis, documents on rheumatism and sequencing could be divided into two major categories, indicated by the blue and red clusters (**Figure S7A, B**). The blue cluster, in which the central word, “biomarker”, is surrounded by the terms “mass-spectrometry”, “proteins”, “plasma”, “synovial fluid”, and “serum”, symbolizes a biomarker-centered topic, in which mass spectrometry is applied to look for novel proteins in plasma, synovial fluid, or serum. The other cluster, colored red, with the word “disease” in the center and surrounded by “criteria”, “classification”, “arthritis”, “association”, “mechanism”, and so on, refers to the diverse but relatively tightly linked aspects of rheumatic diseases.

Centrality refers to the relevance of certain themes to the field, and density refers to the degree of development of these themes. Five groups of themes were scattered on the four-quadrant graph with each quadrant representing different state of themes. Motor themes refer to the ones with high relevance and rapid development, serving as driving force in this field. Basic themes refer to the ones with high relevance and low development, serving as cornerstones in this field. Niche themes refer to the ones with high development and low relevance. Emerging or declining themes refer to the themes with low relevance and low development, which are probably the newly emerged themes or the themes losing its importance and attention (**Figure 7B**). The blue-colored group, comprising the themes “association”, “classification”, and “disease activity”, and the red-colored group, comprising the themes “expression”, “cells”, and “activation”, lay in the motor themes quadrant.. Themes such as “identification”, “rheumatoid arthritis”, and “protein” had solid connections to the filed but lacked development, serving as basic themes. Studies of themes related to “disease”, “risk”, and “susceptibility” were highly developed, despite their relatively weak association with the field of rheumatism and were regarded as niche themes. The themes of “diagnosis”, “biomarkers”, and “serum” and the themes of “senile idiopathic arthritis”, “peripheral blood”, and “I interferon” were placed in the quadrant of emerging or declining themes.

## 4 Discussion

### 4.1 General information

Although studies into rheumatism can be traced back thousands of years, our understanding of the disease was, until recently, severely hampered by its intricate characteristics and lack of efficient tools available for the exploration of its pathogenesis. Complicated clinical manifestations, complex categorizations, and a veiled pathogenesis, which could only be better analyzed with revolutionary technologies, stood in the way of our explorations. Former studies were restricted to analyzing rheumatism on the clinical and semiological levels, without a detailed analysis of the molecular level. Moreover, the critical role of pathogens in rheumatism, such as *Streptococcus pyogenes* in rheumatic fever, also demanded analysis from the molecular level. Fortunately, sequencing technology was developed and brought answers to these long-unsolved problems, as well as drastic changes to the field, as a result of its capability of investigating molecular patterns. Details of the pathological and immunological processes underlying rheumatic diseases were elucidated, more comprehensive disease classifications were designed, and hidden stages of diseases were revealed. Today, sequencing technology undoubtedly serves as a major driving force in studies of rheumatism.

Moreover, the rapid changes in sequencing technology are also altering the trajectory of rheumatism studies. The announcement, on 26 June 2000, that scientists from the Human Genome Project (HGP) had achieved the important milestone of completion of a working draft sequence, ushered in the post-genomics era, which placed more stress on the study of functional genomics, specifically the dynamic genome, and its products such as RNA, proteins, and metabolites (46). In line with this development of sequencing technology, which shifted from DNA sequencing to transcriptomics, then to proteomics, to metabolomics, and to epigenetics, sequencing technology enabled scholars to find genetic patterns of rheumatic susceptibility, its gene expression characteristics, and to apply proteomics to identify novel biomarkers.

In this bibliometric analysis, we examined 1,374 articles published from 2000 to 2022 (April 25) on sequencing technology and rheumatism.

The number of annual publications, to a certain extent, can reflect the level of researchers’ interest in the field. The number of articles on rheumatism and sequencing started to increase significantly after 2000 and has continued to exhibit an overall increase, despite a few ups and downs. In the year 2005, the number of publications saw its first peak, with most publications focusing on proteomics and gene expression profiling. The same theme lasted for years, until the technique of single-cell RNA sequencingbecame mature and was widely deployed, as a result of which it became the most popular research topic in rheumatism and sequencing. Sequencing in rheumatism regained its popularity in 2009, and the number of articles published increased to nearly 100 per year. In 2017, the publication number received another boost, and in 2021 reaching a record high of 152 publications.

Among the 62 countries that dedicated efforts to this field, the USA ranked first, in terms of both the total number of documents and total number of MCPs, and was the home to a large proportion of most relevant affiliations, such as Stanford University, the University of California, San Francisco, and so on. China came in second in total publication numbers, but had the highest number of SCPs. Countries also worked together to conquer obstacles, with the USA having the strongest cooperative bonds with China, European countries, and Korea. Switzerland had the highest MCP ratio, indicating that most of its publications resulted from collaboration with other countries. In terms of the most relevant authors, Li from Shanxi Medical University and Liu from the First Affiliated Hospital of Anhui University of Traditional Chinese Medicine were at the top, with 16 and 15 related articles published in total, respectively, and they are still very active today. Baechler of the University of Minnesota and Gregerson of the Feinstein Institutes for Medical Research reached their peak publishing activity in 2005, and their output has since gradually declined. Several connections can be found between the most relevant authors and affiliations. For instance, Shanxi Medical University, which ranked 12th on the most relevant affiliations, is the institution where Li, the most prolific author, works. This indicates that the most relevant authors also played prominent roles in the establishment of great teams and research environments in this field.

*Arthritis & Rheumatology*, *Annals of the Rheumatic Diseases*, and *Arthritis and Rheumatism* were the three most relevant sources, among which the first two presented an increase in 2013. Although *PLOS ONE* had only an impact factor of 3.752 in 2022, it published 36 relevant articles and had an h-index of 19, so its influence on the subject should not be ignored.

Most cited documents, whether globally cited or locally cited, served as milestones for crucial discoveries and as important stimuli of research. For instance, “Complete genome sequence of an M1 strain of *Streptococcus pyogenes*” (32), by Ferretti, published in 2001, mapped out the complete genome sequence of a strain of the pathogen that is responsible for rheumatic fever. This work has greatly increased our understanding of rheumatic fever, which still remains uncontrolled in some countries and regions (47). This work also motivated the study “Genome sequence and comparative microarray analysis of serotype M18 group A *Streptococcus* strains associated with acute rheumatic fever outbreaks” (33), by Smoot, published in 2002. Another perspective on the human immune system response to rheumatic disease is provided by the article “Interferon-inducible gene expression signature in peripheral blood cells of patients with severe lupus” (39), by Baechler, published in 2003. This article, in which Baechler identified IFN pathway dysregulation as a marker of more severe disease in SLE patients, played a central role in the field of SLE research, not only cracking the pathogenesis of SLE but also providing insight into the treatment for it. Although this article was not on the list of either the most globally or locally cited documents, it ranked second on the most locally cited references, revealing its significant impact. Together with “Microarray analysis of microRNA expression in peripheral blood cells of systemic lupus erythematosus patients” (48), by Dai, published in 2007, the third most globally cited document, it is apparent that the peripheral blood acts as an essential substrate for studying the pathogenesis of SLE. From this analysis, two lines of inquiry could be identified in these publications. One focused on the pathogens that might lead to rheumatic disorders, while the other focused on exploring the inner characteristics and immunological responses of rheumatic diseases. Altogether, it can be seen that the focus in the field was transferred from genome sequencing to gene expression profiling and to proteomics. This trend synchronizes with the central dogma of molecular biology and the advancement of sequencing technology.

### 4.2 Hotspots and frontiers

“Disease” and “expression” were the most frequent keywords identified. In addition to these, “rheumatoid arthritis”, one of the most popular diseases studied in rheumatism, accounted for 5% of total word occurrences. Keywords concerning pathogenesis, such as “inflammation”, “activation”, “cells”, and “pathogenesis” itself, were also high in frequency. “TNF-alpha”, "NF-kB", and “tumor necrosis factor” could also be seen in the word cloud, suggesting that there was an emphasis on the study of immunological molecules. In the co-word analysis, keywords were divided into five distinct clusters.

#### 4.2.1 Cluster 1: Rheumatism susceptibility and its related genetic patterns

Cluster 1 was colored in blue, and included the terms “disease”, “susceptibility”, “risk”, “association”, “arthritis”, and “systemic lupus erythematosus”. The word “association” appeared in the phrases genome wide association studies and “genetic association”, aiming to find the correlation or association between single nucleotide polymorphisms (SNPs) and diseases, to estimate the possible risks or susceptibility. For instance, using large-scale GWASs, researchers identified the significant genetic variants connected with rheumatoid arthritis susceptibility (49). Nicotinamide adenine dinucleotide synthetase 1 (*NADSYN1*) and transcription factor 7 (*TCF7*), novel SLE susceptibility genes, were uncovered by GWASs and expression quantitative trait loci (eQTL) analysis (50). All words in this cluster were related to the role of sequencing technology in identifying the genetic characteristics that indicate a risk of developing rheumatism.

#### 4.2.2 Cluster 2: The molecular features of rheumatic disorders

Cluster 2 was colored in red, and consisted of the terms “expression”, “identification”, “gene”, “protein”, “cartilage”, and “articular-cartilage”, concentrating on the molecular basis of rheumatism. More and more relevant molecules, whether genes or proteins, have been found, using sequencing technologies such as microarray, to play a pivotal role in rheumatic disorders. Take SLE as an example: the etiology of SLE is multifactorial, involving genes, epigenetic factors, and autoantibodies (51). Utilizing DNA microarray and extensive microarray, SLE-associated genes and the interferon signature were identified. Applying autoantigen microarray, studies achieved autoantibody profiling in SLE, with the identification of autoantibodies specifically associated with different manifestations (52). The discovery of these molecules helped to decipher the molecular pathways of rheumatic diseases. In addition, the appearance of the words “cartilage” and “articular-cartilage” suggested the emerging appliance in osteoarthritis and rheumatoid arthritis, in which the cartilage is the main site of destruction (53).

#### 4.2.3 Cluster 3: Immunological reaction and pathogenesis

Cluster 3 was colored in orange, with the words “cell”, “pathogenesis”, “inflammation”, and “activation” at its core, implying a pathological and immunological standpoint. Inflammation is an essential part of rheumatism pathology, involving immunology cells, cytokines, and tissue cells. The improvement of single-cell RNA sequencing technology allowed analysis of heterogeneity between cells, in turn identifying key cells in the pathological process. Li et al., utilizing single-cell sequencing among chondrocytes and fibroblasts in osteoarthritis (OA), identified the focal adhesions pathway and two marker genes [i.e., collagen type VI alpha 3 chain (*COL6A3*) and actin gamma 1 (*ACTG1*)] as key components of OA (54). Using bulk and single-cell transcriptomic data analysis, Lee EJ et al. identified the pathways that are significantly activated in RA, such as the Th1 pathway, interferon signaling, and CDC42 signaling, and their differential expression pattern of expression between tissues (synovium, white blood cells, peripheral blood mononuclear cells, and CD4+ T cells) (55). Rossetti M et al. conducted T-cell receptor sequencing and found clonotypes shared between blood and synovial fluid, suggesting that inflammation is associated with Treg cells’ recirculation in RA patients (56). Interestingly, the word “cancer” also according to several findings (57, 58), cancer and rheumatic disorders share similar biological pathways. For instance, CCL20-CCR6 axis appears both in RA and cancer progression (59). Hypomethylation, resulting in stimulation of interspersed repetitive sequences (IRSs), such as LINE-1 and Alu, could trigger rheumatic diseases as well as cancer (59). In addition, metabolomics studies have shown that cancer and inflammation share similar metabolic characteristics (58). Altogether, accumulating evidence shows that sequencing technology has a huge impact on unraveling the pathological progression of rheumatic diseases.

#### 4.2.4 Cluster 4: Categories and stages of rheumatic diseases

Cluster 4, colored in green, mainly consisted of the terms “classification” and “disease-activity”. One prominent characteristic of rheumatic diseases is the overwhelming complexity of their categorization. The complex clinical manifestations and confusing disease activity of rheumatic diseases, such as different stages of disease progression or activation, made classification a tricky problem. However, with the help of sequencing, we were able to categorize them with more precision. Interferon signature genes, which have high expression levels in active SLE, were found to signify disease progression and activity in patients with incomplete lupus syndrome (ILE) and refined our classification standards for SLE (60). Ambiguity in juvenile idiopathic arthritis (JIA) classification was also clarified with the assistance of high-throughput omics technologies, which identified a number of human leukocyte antigen (HLA) alleles and 23 non-HLA genetic loci associated with different JIA phenotypes (61). Metabolomics analysis also assisted the division of osteoarthritis into subgroups with different metabolic activity (62). Undoubtedly, sequencing technology has greatly improved our understanding of the classification and progression of rheumatic disorders.

#### 4.2.5 Cluster 5: Novel biomarkers for early diagnosis

Cluster 5 was colored purple, with the term “rheumatoid arthritis” in the center, surrounded by the terms “diagnosis”, “biomarkers”, “osteoarthritis”, and “mass-spectrometry”. According to the 2010 American College of Rheumatology/European League Against Rheumatism (ACR/EULAR) classification criteria (63) for RA’s diagnosis standards, the focus of RA’s diagnosis was placed more on the early stages of the disease course, during which biomarkers could be examined through laboratory tests. This was in huge contrast to the 1987 version, which focused more on the clinical symptoms that appear late in disease progression (64). Biomarkers, often refer to detectable molecules that can signal changes or disease in tissues, cells, or subcellular components, serve as effective indicators for rheumatic diseases. They can often be discovered by mass spectrometry of serum, plasma, or synovial fluids. For example, Mun S et al. conducted mass spectrometry and validated constitutive serum amyloid A4 (*SAA4*), gelsolin (*GSN*), and vitamin D-binding protein (*VDPR*) as RA biomarkers, which has value for not only the diagnosis but also the prevention and treatment of rheumatic diseases (65). He ZR et al. applied gas chromatography–mass spectrometry (GC/MS) and found a three-metabolite marker panel that contains L-cysteine, citric acid, and L-glutamine, giving detailed insights into the pathological process of RA (66). Similarly, in osteoarthritis, biomarkers are also of great use. Chen R et al. distinguished that alanine, gamma-aminobutyric acid, and 4-hydroxy-L-proline are biomarkers that separate OA patients (41). The discovery of new biomarkers is significant not only because it improves our understanding of the biochemical or cellular mechanisms of rheumatism but also because biomarkers have great clinical prospects, with more and more researchers studying the laboratory examinations and diagnostics of rheumatic diseases.

Although keywords were separated into five clusters, cross-links between clusters still existed. Exploring the genetic expression pattern could also contribute to an understanding of an individual’s susceptibility to rheumatic diseases, as well as their disease classification status. Discovering novel biomarkers could also lead to the further explication of the pathological or physiological progress involved in rheumatism. Thus, it is of great importance to bear in mind the close relationship between these key themes. In addition, of the various diseases that rheumatism covers, it is apparent that RA, SLE, and OA are the hottest spots for research, with solid foundations and great clinical significance. All in all, sequencing technology was introduced to rheumatism studies to find solutions for unsolved problems, but it is now altering and even leading groundbreaking studies in rheumatism.

Nevertheless, limitations in our study still exist. Our study included only articles published from 2000 to 2022, which might have led to the oversight of both early important studies and the latest updates.

## 5 Conclusion

In this bibliometric study, we reviewed the progress made in the last 22 years in sequencing and rheumatology, and identified the hotspots and frontiers for future investigation. Evidently, sequencing technology has invigorated the study of rheumatism. From the standpoint of pathogenesis, genomic sequencing has equipped us with knowledge of the pathogens involved, and identified possible genetic backgrounds and molecules involved in the pathogenesis of rheumatism. From the standpoint of clinical value, genomic sequencing has identified possible markers for diagnosis, supplemented evidence for defining stages and classifications, and spotted potential targets for therapy. Among those contributions, employing sequencing technology to study the pathogenesis and classifications of rheumatism would be a wonderful direction for future studies to take. Discovering novel biomarkers for diagnosis and uncovering genetic patterns related to disease susceptibility are also promising orientations. We hope that, with this bibliometric study, we can provide a reference for researchers to better grasp the trends and key points of the study of rheumatism.

## Data availability statement

The original contributions presented in the study are included in the article/**Supplementary Material**, further inquiries can be directed to the corresponding authors.

## Author contributions

Conception/design: RH, JT, SW, YLiu, MZ, MJ, HQ, WQ, YLu, YiY, BL, YuY, PY, JH, WZ, JL, MG, YZ, XG, SX, XL, and ZH. Collection and/or assembly of data: RH, JT, SW, YLiu, MZ, MJ, HQ, WQ, YLu, YY, BL, YY, PY, JH, WZ, JL, MG, YZ, XG, SX, XL, and ZH. Data analysis and interpretation: RH, JT, SW, YLiu, MZ, MJ, HQ, WQ, YLu, YiY, BL, YuY, PY, JH, WZ, JL, MG, YZ, XG, SX, XL, and ZH. Manuscript writing: RH, JT, SW, YLiu, MZ, MJ, HQ, WQ, YLu, YiY, BL, YuY, PY, JH, WZ, JL, MG, YZ, XG, SX, XL, and ZH. Final approval of manuscript: RH, JT, SW, YLiu, MZ, MJ, HQ, WQ, YLu, YiY, BL, YuY, PY, JH, WZ, JL, MG, YZ, XG, SX, XL, and ZH. All authors contributed to the article and approved the submitted version.
