# Supplementary material for: Sequencing technology as a major impetus in the advancement of studies into rheumatism: A bibliometric study
Source: Front Immunol. 2023 Feb 17;14:1067830. doi: 10.3389/fimmu.2023.1067830 (PMC9982012; doi:10.3389/fimmu.2023.1067830)
Supplement: Supplementary file 1 [file DataSheet_1.docx]

**Supplementary Figures**


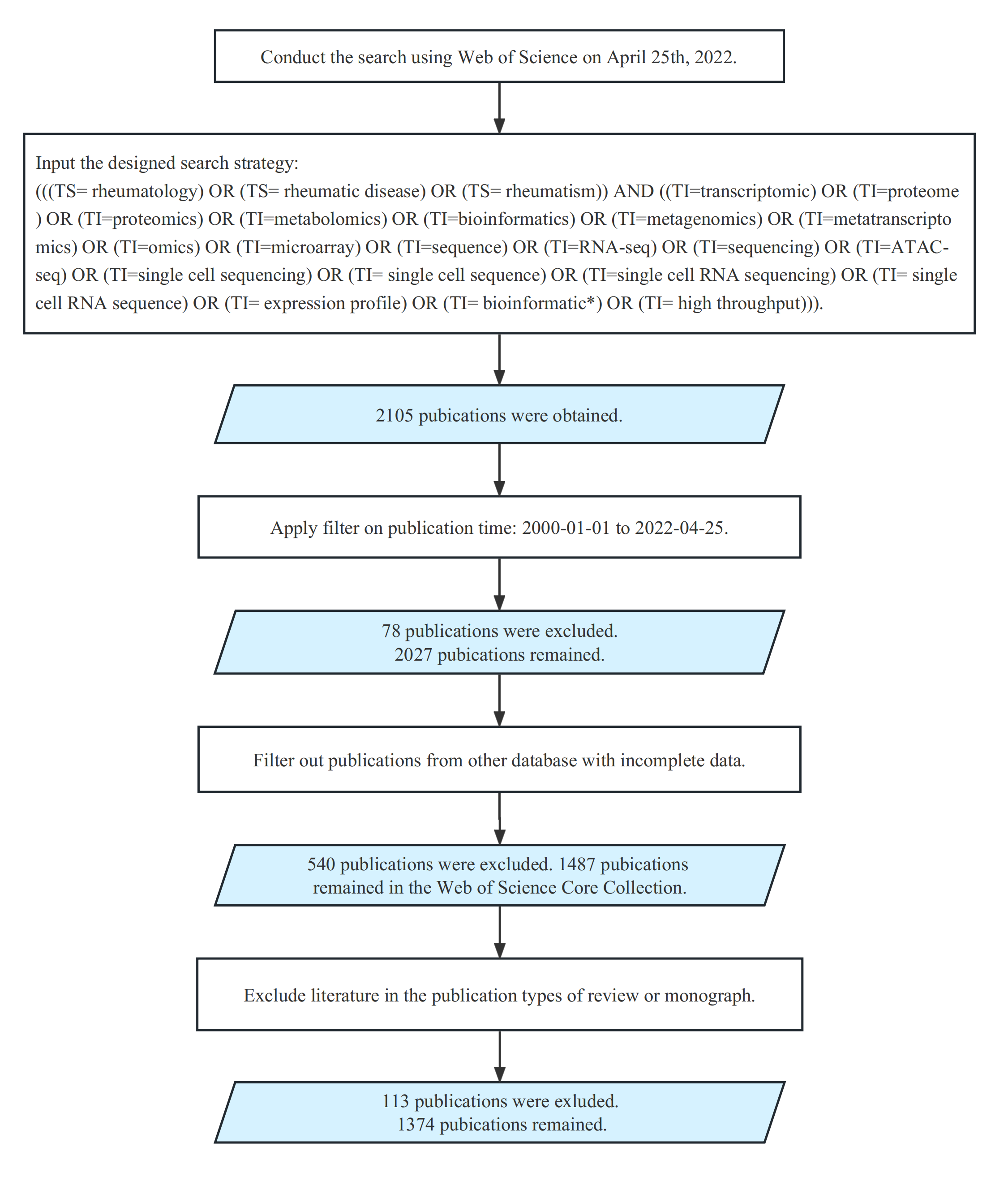


Figure S1：Flowchart of inclusion and exclusion criteria for relevant articles.

Figure S2: Supplementary for figure 2

1. Top 20 most cited countries. Diameter and color darkness of the node was in proportion to the number of documents published by the country or region.
2. Corresponding author’s countries. SCP: Single country publication; MCP: multiple countries publications.
3. The frequency distribution of scientific productivity.
4. Top 20 authors’ production over time. Red line represented the length of years when the author published related articles. Color darkness and size of the node was in proportion to the number of documents published by the author.


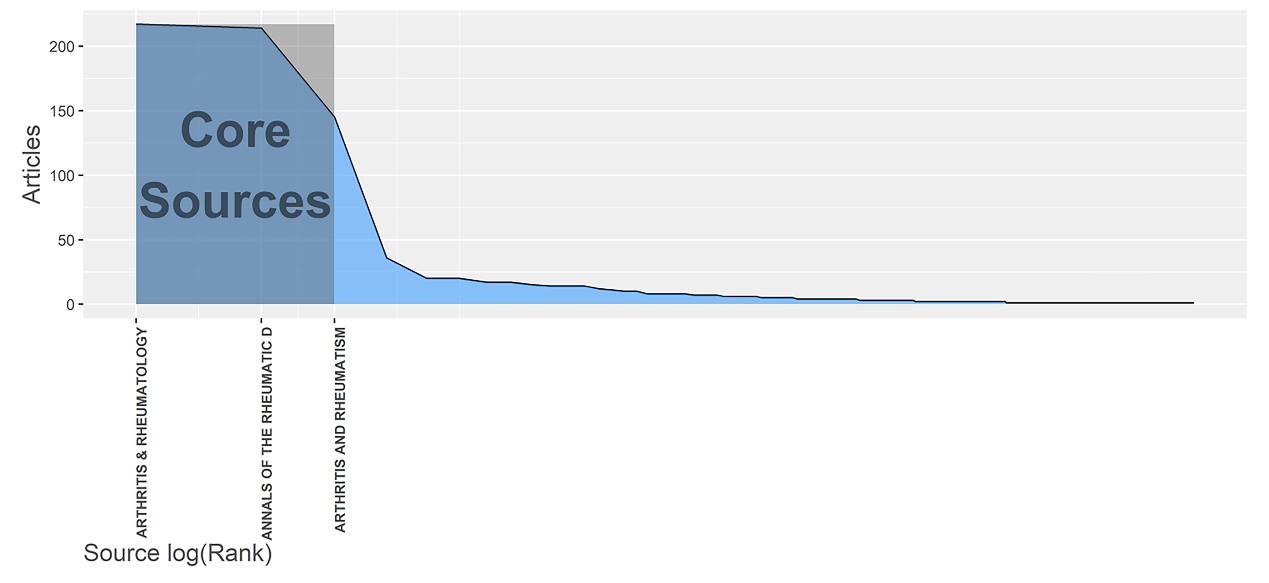


Figure S3: Core sources analysis.

The horizontal axis was labeled sources related with rheumatism and sequencing. The vertical axis represented the number of articles published on each source. Core sources included Arthritis & Rheumatology, Annals of the rheumatic diseases, Arthritis and rheumatism.


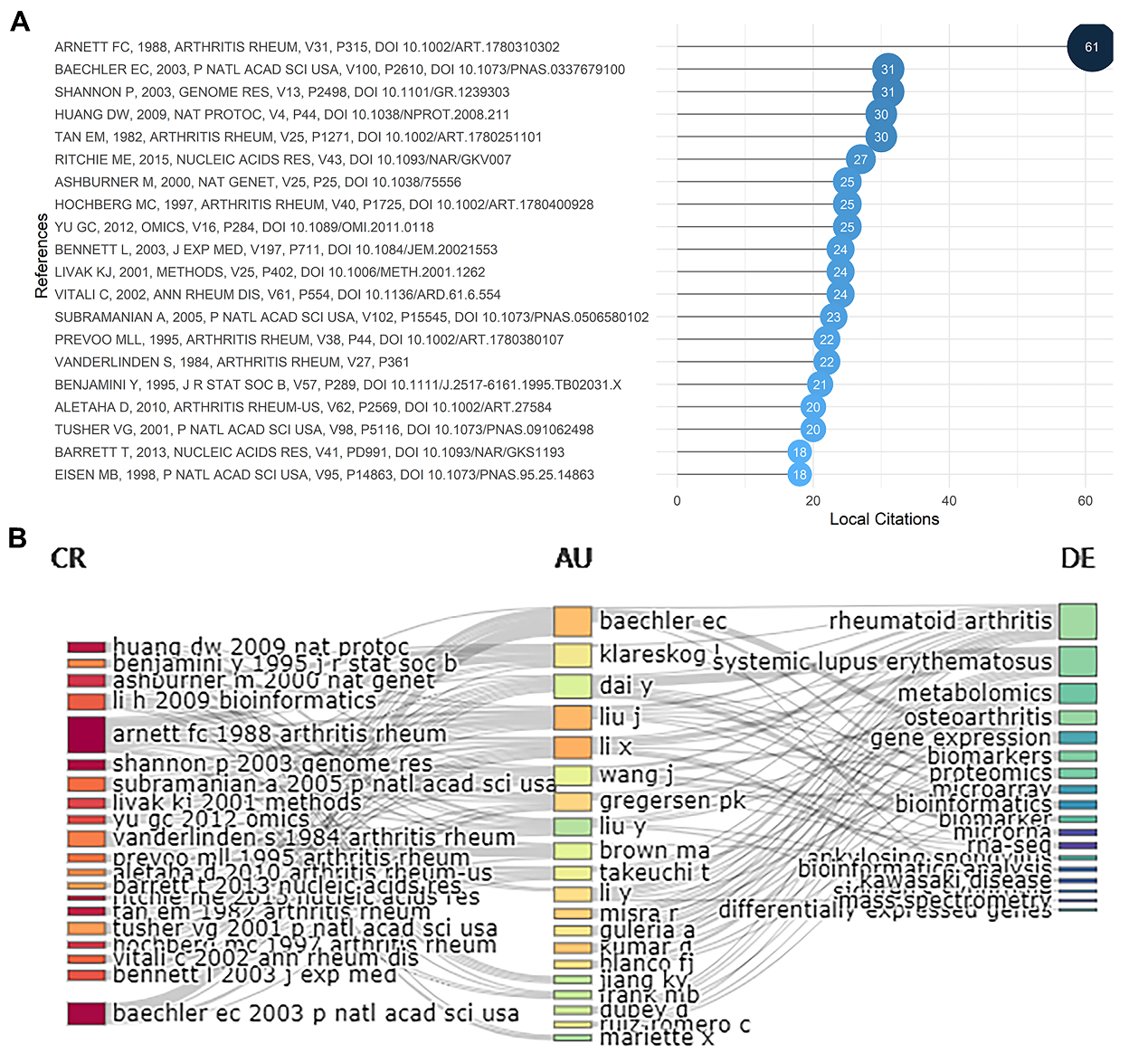


Figure S4：Supplementary figures for figure 4.

A. Top 20 most local cited references. Size and darkness of the nodes were in proportion to the citation number of each reference.

B. Sankey diagram of cited references, authors, and author’s keywords.


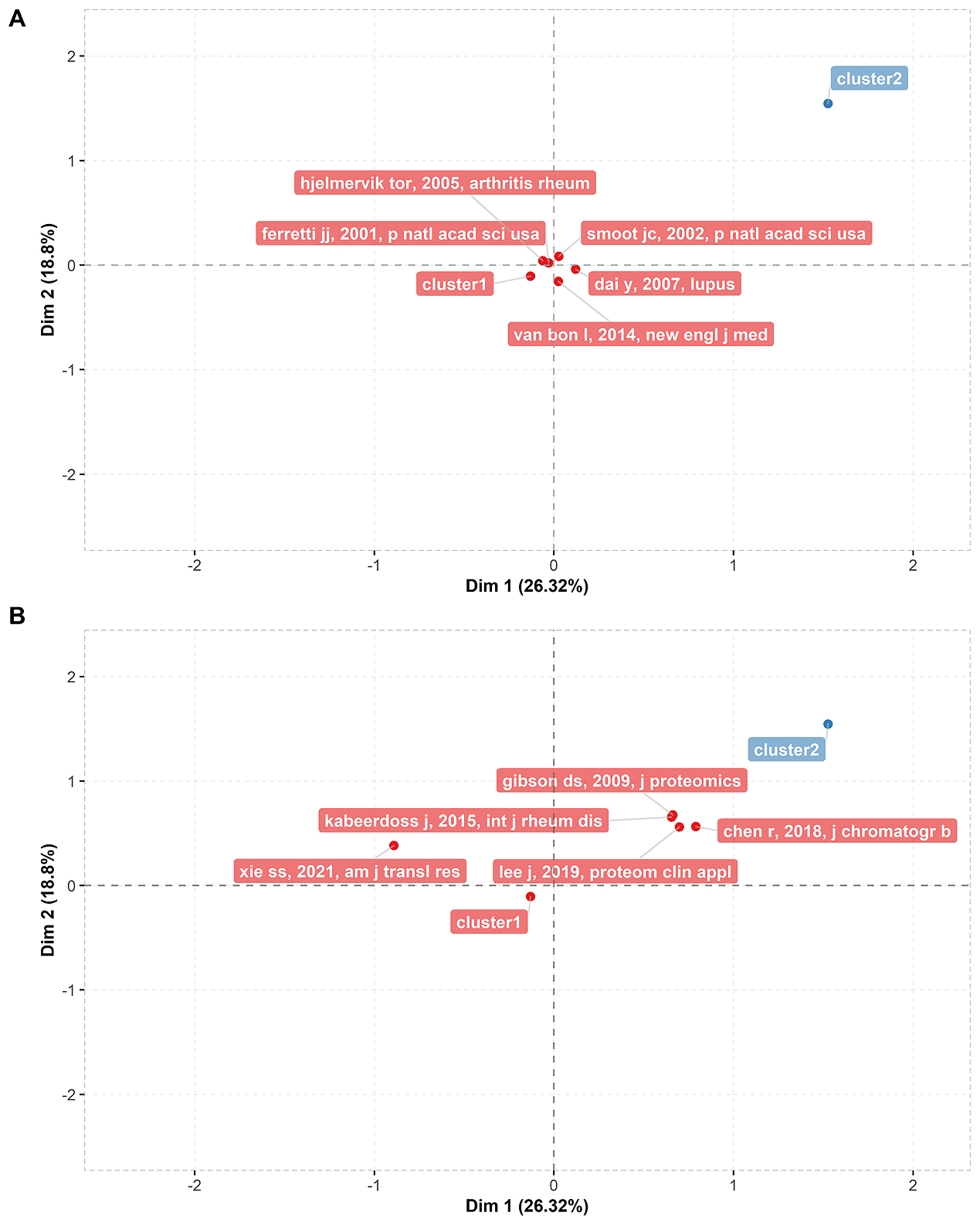


Figure S5: Factorial analysis of relevant documents.

1. Factorial map of the most cited documents
2. Factorial map of the documents with the highest contributes

Figure S6: Supplementary for figure 5.

1. Keyword tree of keywords selected from Keyword Plus. Square’s size represented keyword’s occurrences compared to the whole occurrences.
2. Keyword Cloud. The size of the words represented the occurrence frequency of the keyword.


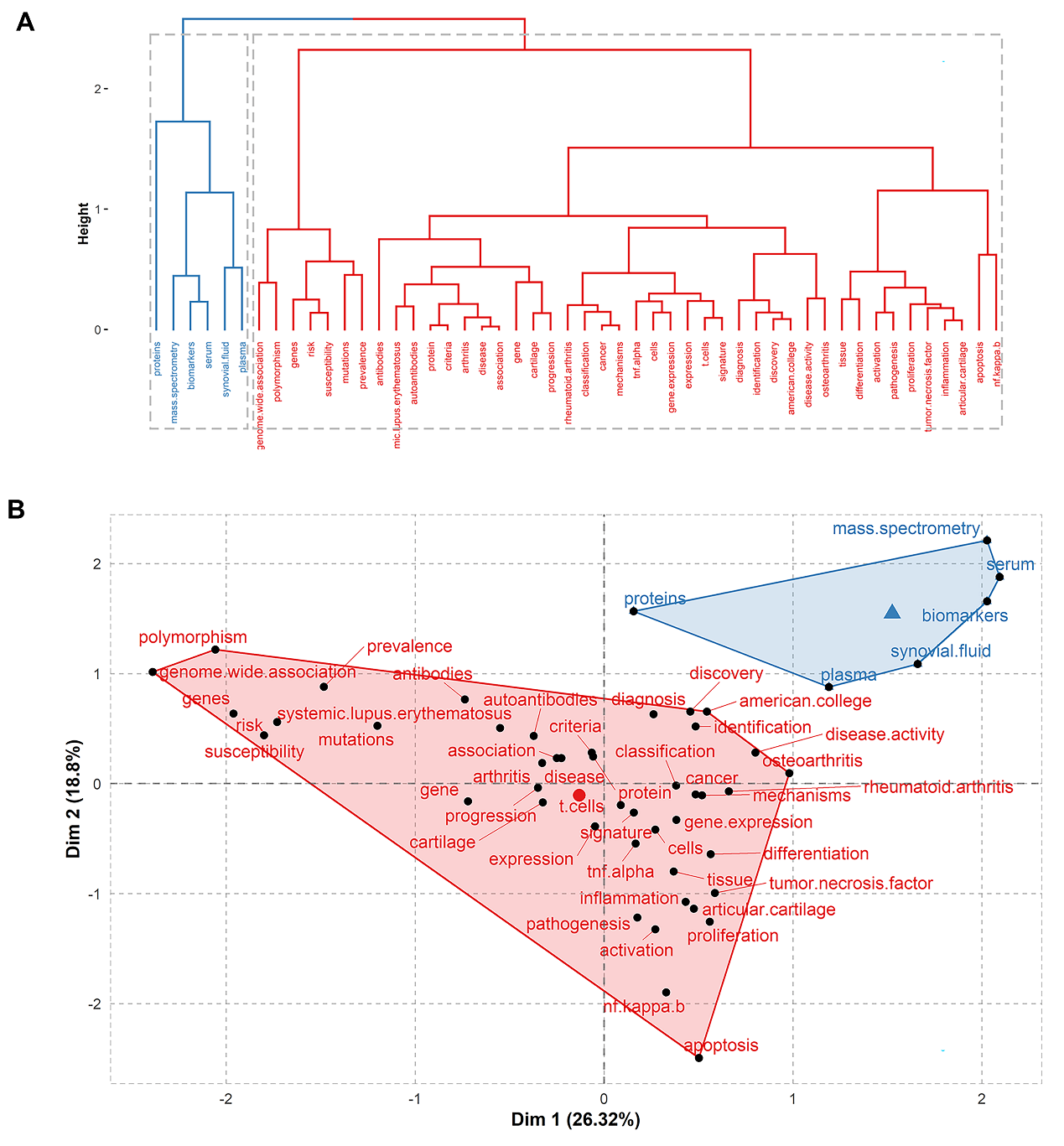


Figure S7: Supplementary for figure 6.

1. Topic dendrogram. Topics that shared close relation were closer on the dendrogram. Two main branches were respectively labeled blue and red.
2. Conceptual structure map of themes using MCA. Topics that shared close relation were closer on the map. Two main groups were respectively colored blue and red.
